# Decoding the role of RPL38 in lung adenocarcinoma: a multi-omics approach

**DOI:** 10.3389/fimmu.2026.1778481

**Published:** 2026-02-12

**Authors:** Lu Zhang, Fei Teng, Yuan Wang, Yang Chen, Qiuxia Liu, Fengsheng Dai, Liang Yu, Chenguo Yao, Zhiqiang Wang

**Affiliations:** 1 Center of Thoracic Cancer, Chongqing University Caner Hospital, Chongqing, China; 2 Chongqing Key Laboratory for the Mechanism and Intervention of Cancer Metastasis, Chongqing, China; 3 The State Key Laboratory of Power Transmission Equipment and System Security and New Technology, School of Electrical Engineering, Chongqing University, Chongqing, China

**Keywords:** lung adenocarcinoma, multi-omics data, progression, RPL38, tumor microenvironment, tumor immune

## Abstract

**Introduction:**

Members of the Ribosomal Protein L (RPL) family are involved in diverse biological processes and cancer biology, yet their precise functions and clinical implications in lung adenocarcinoma (LUAD) remain incompletely understood.

**Methods:**

Machine learning was applied to The Cancer Genome Atlas (TCGA) data to identify pivotal RPL genes and construct a predictive risk model. Multi-omics analyses—including pan-cancer cohorts and spatial transcriptomics—were integrated to evaluate the expression and prognostic significance of Ribosomal Protein L38 (RPL38). Functional impacts were examined using CCK‑8, colony formation, wound healing, Transwell assays, and subcutaneous xenograft models.

**Results:**

A three‑gene RPL‑based prognostic signature was established from the TCGA‑LUAD cohort. High‑risk patients exhibited shorter survival and increased immunosuppressive characteristics. RPL38 was upregulated in multiple cancers and associated with unfavorable outcomes. Immunohistochemical and spatial transcriptomic analyses confirmed its aberrant expression in LUAD and linked it to an immunosuppressive tumor microenvironment. Genetic ablation of RPL38 significantly inhibited LUAD cell proliferation and migration in vitro, and impaired xenograft tumor growth in vivo.

**Conclusions:**

RPL38 plays a tumor‑promoting role in LUAD. This study clarifies the contribution of RPL38 to LUAD development, provides new insights into its pathogenesis, and suggests a rationale for therapeutic targeting of RPL38 in LUAD treatment.

## 1 Introduction

As the most common histologic subtype of non-small cell lung cancer (NSCLC), lung adenocarcinoma (LUAD) is a leading cause of global cancer mortality (1, 2). Diagnostic outcomes have improved with the adoption of early screening techniques like low-dose computed tomography (LDCT); however, many patients are still diagnosed at advanced stages, which significantly limits curative options (3, 4). Current treatment protocols extend beyond traditional platinum-based chemotherapy to include molecularly targeted therapies and immune checkpoint inhibitors, substantially improving outcomes for selected populations. Yet, the development of resistance to these treatments remains a critical clinical challenge (5–8). The integration of multi-omics data—encompassing genomic, transcriptomic, and proteomic dimensions—has advanced the molecular classification of LUAD, enabled more personalized therapeutic approaches, and identified potential new biomarkers for early detection and prognosis evaluation.

Within the large ribosomal subunit, ribosomal protein L (RPL) family members function as core structural and catalytic constituents, primarily recognized for their indispensable role in protein translation (9, 10). Emerging studies, however, highlight the participation of these proteins in a spectrum of functions outside the ribosome, including key events in oncogenesis and malignant progression (11, 12). Dysregulation of RPL genes is a common feature in numerous malignancies and often correlates with patient prognosis. As an example, RPL11 can interact with MDM2 to inhibit its function, leading to the stabilization of p53 and the initiation of an anti-tumor response—a regulatory axis frequently disrupted in cancer (13, 14). Conversely, proteins like RPL15 and RPL34 have been shown to promote tumorigenesis and resistance to chemotherapy through the activation of pro-oncogenic signaling cascades (15–17). Furthermore, the abundance of particular RPLs can serve as a standalone prognostic marker, where higher expression levels are predictive of reduced survival in cancers such as those affecting the breast, lung, liver, and colon (16–20). Despite these insights, the specific roles and clinical relevance of the RPL family in the context of LUAD are not yet well defined.

This study obtained the list of RPL family members from the HGNC database. Differentially expressed RPL proteins in LUAD were identified by intersecting this list with differentially expressed genes (DEGs) from the TCGA-LUAD dataset. Subsequent Cox proportional hazards analysis pinpointed three central genes—RPLP0, RPL39L, and RPL38—leading to the construction of a prognostic risk model using the eXtreme Gradient Boosting (XGBoost) algorithm. A particular focus was placed on RPL38, an RPL family member exhibiting significant upregulation in LUAD whose functional role was previously undefined. By integrating bulk RNA sequencing with spatial transcriptomic data, we characterized the expression and mutational profiles of RPL38 and investigated their relationship with the tumor microenvironment (TME). The biological function of RPL38 in LUAD was further evaluated through a series of *in vitro* and *in vivo* experiments. In summary, this research employs a multi-omics integrative strategy to clarify the clinical significance of RPL38, providing new insights relevant to the diagnosis, therapeutic intervention, and prognosis of LUAD.

## 2 Methods

### 2.1 Acquisition and processing of raw data

The list of genes encoding ribosomal protein L (RPL) family members was retrieved from the HGNC database (https://www.genenames.org/; see Additional file 1). RNA sequencing data, along with clinical annotations and somatic mutation information from the TCGA-LUAD cohort, were downloaded from the UCSC Xena platform (https://xena.ucsc.edu/). Differential gene expression analysis between tumor and adjacent normal tissues was conducted using the DESeq2 package (21) in R, with thresholds set at FDR < 0.05 and |log_2_FC| > 0.5. This analysis identified 9247 significantly dysregulated genes, comprising 5736 up-regulated and 3511 down-regulated genes in tumors relative to normal tissues (Additional file 2).

### 2.2 Development of an RPL-based prognostic model

By taking the intersection between the RPL gene set and the TCGA-LUAD DEGs via the “VennDiagram” R package, 15 overlapping genes were obtained. Univariate Cox regression was used to identify three prognosis-relevant RPL genes. A predictive risk model was constructed based on these genes using the XGBoost algorithm. Cases in the TCGA-LUAD dataset with available survival information were randomly divided into training and validation subsets at a 7:3 ratio using a fixed random seed (seed = 993). The training subset was used for model construction, with key parameters set as follows: maximum tree depth = 6, learning rate (eta) = 0.5, and number of boosting rounds = 10, while the validation subset was used to evaluate predictive performance.

### 2.3 Assessment of immune infiltration and escape

The “xCell” R package was employed to estimate immune cell enrichment in high- and low-risk patient groups. The potential for immune escape was further evaluated using the TIDE platform (http://tide.dfci.harvard.edu/), which computes composite scores related to TIDE, immune dysfunction, exclusion, and MDSC activity.

### 2.4 Analysis of drug sensitivity

Drug response data were obtained from the GDSC resource (https://www.cancerrxgene.org/). The “oncoPredict” R package, specifically the calcPhenotype function, was used to infer individual drug sensitivity profiles for each sample.

### 2.5 Processing of spatial transcriptomic data

The spatial transcriptomics dataset GSE179572 was analyzed to examine the tissue-level expression distribution of key genes. Profiling of LUAD sections was carried out using the Sparkle system (https://www.grswsci.top).

### 2.6 Cell line culturing

Human LUAD cell lines A549 and H1299 were procured from Pricella Biotechnology (Wuhan, China). Cells were grown in RPMI-1640 medium (Gibco, USA) supplemented with 10% FBS (Gibco) and 1% penicillin/streptomycin (Gibco), under standard culture conditions (37 °C, 5% CO_2_). All cell identities were confirmed via short tandem repeat (STR) profiling.

### 2.7 Protein expression analysis via western blot

Western blotting was carried out based on previously described methods (22). Antibodies used in this study included: rabbit monoclonal anti-RPL38 (Proteintech, 15055-1-AP; 1:1000), mouse monoclonal anti-β-actin (Proteintech, 66009-1-Ig; 1:10,000), HRP-conjugated goat anti-mouse secondary antibody (Proteintech, RGAM001; 1:10,000), and HRP-conjugated goat anti-rabbit secondary antibody (Proteintech, RGAR001; 1:10,000).

### 2.8 Establishment of stably transfected cell lines

Lentiviral vectors expressing short hairpin RNAs targeting RPL38 (sh-RPL38) and a non-targeting control (shNC) were purchased from Genechem Co., Ltd (Shanghai, China). The specific shRNA sequences were: sh-RPL38#1: AAAAGGACAACGTGAAGTTTAAATTGGATCCAATTTAAACTTCACGTTGTCC; sh-RPL38#2: AAAAGCCTCGGAAAATTGAGGAATTGGATCCAATTCCTCAATTTTCCGAGGC. A549 and H1299 cells were plated in 6-well plates and transduced at approximately 20–30% confluence. Following a 72-hour incubation, stable integrants were selected with puromycin (1 μg/mL, Beyotime, #ST551) for one week. Successful knockdown was confirmed by western blot in both cell lines.

### 2.9 Evaluation of cellular proliferation

For CCK-8 assays, stable A549 and H1299 cells were seeded into 96-well plates (1.5 × 10³ cells/well). Cell viability was measured at 0, 24, 48, 72, and 96 hours by adding 10 μl of CCK-8 reagent (Abbkine, #BMU106-CN) per well, followed by a 2-hour incubation in the dark. Each measurement was performed in triplicate.

In colony formation experiments, cells were seeded in 6-well plates (1 × 10³ cells/well) and cultured for 14 days, with the medium replaced every 7 days. Colonies were fixed with 4% paraformaldehyde (Biosharp, #BL539A), stained with 0.5% crystal violet (Phygene, #PH1322), imaged, and counted. The assay was repeated three times independently.

### 2.10 Analysis of migration and invasion

Scratch wound healing assays were conducted using A549 and H1299 cells grown to full confluence in 6-well plates. Cells were serum-starved for 12 hours, and then a straight scratch was made with a sterile 200 μl pipette tip. After washing, cells were maintained in medium with 2% FBS. Wound closure was recorded at 0 and 24 hours and quantified with ImageJ software. Each condition was tested in triplicate.

Transwell assays were used to assess migration and invasion. For migration, cells were seeded into the upper chamber of uncoated Transwell inserts (CORNING, #3422). For invasion, inserts were pre-coated with Matrigel (BD Biosciences). A549 cells (4×10^4^) or H1299 cells (5×10^4^) in serum-free medium were added to the upper chamber, while the lower chamber contained medium with 20% FBS. Following incubation (24 hours for migration, 48 hours for invasion), cells on the lower surface were fixed, stained with crystal violet, and counted under an inverted light microscope (Leica). All assays were performed in triplicate.

### 2.11 *In vivo* subcutaneous tumor model

Animal studies were approved by the Animal Ethics Committee of Chongqing University Cancer Hospital (Approval No. CQCH-LAE-A0000202094). A total of five 4-week-old female nude mice (BALB/c-nu, Gem Pharmatech Co., Ltd) were housed under pathogen-free conditions. Each mouse received bilateral subcutaneous injections of 5×10^6^ A549 cells (either sh-RPL38#1 or sh-NC) into the axillary area. Tumor sizes were measured every other day from day 7 post-injection, and volumes were calculated as V = (length × width²)/2. On day 29, mice were euthanized by gradual displacement of chamber air with compressed CO_2_ at a flow rate of 30-40% of the chamber volume per minute, followed by confirmation of death by cervical dislocation. Tumors were then harvested and weighed.

### 2.12 Statistical methods

All statistical analyses and graphical representations were performed using R software (v4.2.1) or the XianTao platform (https://www.xiantao.love/). For comparisons of continuous variables between two groups, we first assessed the distribution characteristics of the data: normality was verified using the Shapiro-Wilk test, and homogeneity of variances was assessed using Levene’s test. If the data satisfied both normality and homoscedasticity, parametric tests (Student’s t-test) were applied; otherwise, non-parametric tests (Wilcoxon rank-sum test) were used. Comparisons of categorical variables were performed using the Chi-square test or Fisher’s exact test. Survival differences were analyzed by the log-rank test. Correlation analyses employed Pearson’s or Spearman’s methods based on data distribution. A p-value below 0.05 was considered statistically significant. The specific test used in each analysis is indicated in the corresponding figure legend.

## 3 Results

### 3.1 Development and validation of an RPL-based prognostic model in LUAD

#### 3.1.1 Development of the prognostic model

**Figure 1:**
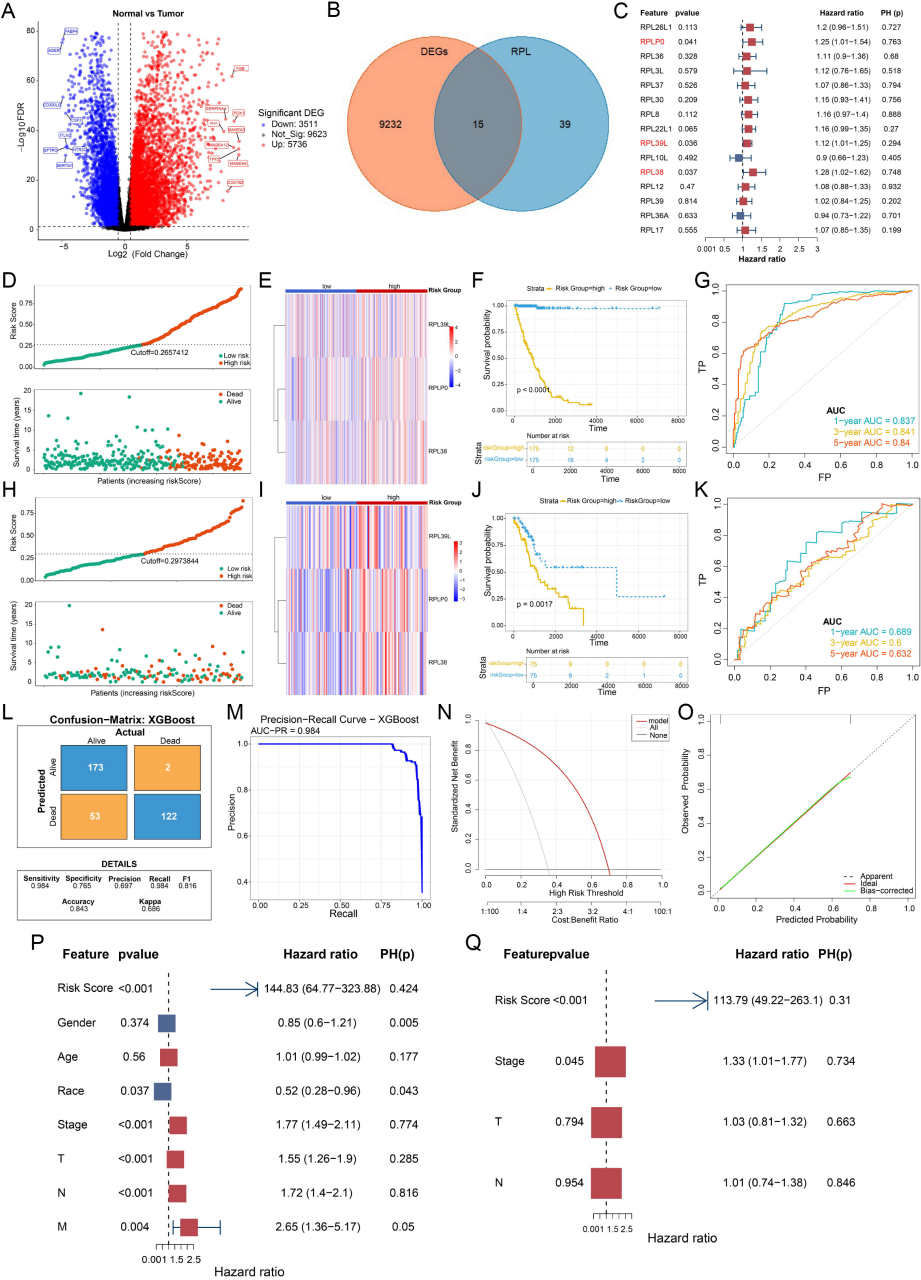
Development of an RPL-derived prognostic signature for LUAD. **(A)** Volcano plot shows the distribution of mRNA expression differences between LUAD tumors and matched normal tissues. **(B)** Venn diagram identifies overlapping genes. Differentially expressed genes are in orange, RPL family genes in blue, and the intersection represents shared candidates. **(C)** Forest plot from univariate Cox proportional hazards analysis of candidate RPL genes. **(D)** Training cohort: distribution of patient risk scores and associated survival status (alive, green; deceased, red), grouped by high or low risk. **(E)** Expression heatmap of the three signature genes across risk groups in the training set. **(F)** Kaplan–Meier curves for overall survival in the training cohort, comparing high- and low-risk groups. **(G)** Time-dependent ROC curves at 1, 3, and 5 years for the training set. **(H)** Validation cohort: risk score distribution and corresponding survival status, stratified by risk. **(I)** Heatmap showing expression of the three key genes in the validation set, grouped by risk category. **(J)** Kaplan–Meier survival analysis for the validation cohort based on risk stratification. **(K)** ROC curves assessing 1-, 3-, and 5-year predictive performance in the validation set. **(L)** Confusion matrix evaluating the classification accuracy of the prognostic model. **(M)** Precision-Recall (PR) curve for model performance. **(N)** Decision curve analysis (DCA). The x-axis shows the threshold probability, and the y-axis the net benefit. Curves represent strategies: classifying all as deceased (“ALL”), all as alive (“NONE”), or using the model’s prediction (“Model”). **(O)** Calibration plot for the prognostic risk model. **(P)** Forest plot from univariable Cox regression analysis of prognostic factors. **(Q)** Forest plot from multivariable Cox regression analysis of prognostic factors.

Comparative analysis of RNA-seq data for tumor and adjacent normal tissues from the TCGA-LUAD cohort enabled the identification of 9247 differentially expressed genes (DEGs) (**Figure 1A**). Cross-referencing these DEGs with the list of 54 known ribosomal protein L (RPL) family members resulted in the isolation of 15 RPL-related DEGs (RPL-DEGs) (**Figure 1B**). To evaluate their prognostic impact, a univariate Cox proportional hazards analysis was conducted on these 15 candidates (including RPL26L1, RPLP0, RPL36, RPL3L, RPL37, RPL30, RPL8, RPL22L1, RPL39L, RPL10L, RPL38, RPL12, RPL39, RPL36A, RPL17) within the TCGA-LUAD dataset. The analysis designated RPLP0, RPL39L, and RPL38 as significant risk factors, each harboring a hazard ratio (HR) greater than 1 (**Figure 1C**). Enhanced expression of these three genes was consistently observed in tumor specimens at both transcriptional and translational levels (**Supplementary Figures S1A, B**), and Kaplan–Meier survival plots confirmed a substantial correlation between their expression and shorter overall survival (OS) in LUAD patients (**Supplementary Figure S1C**).

A predictive model was subsequently constructed by applying the eXtreme Gradient Boosting (XGBoost) algorithm to the expression profiles of these three genes. Using patient survival status (alive/deceased) as the target, the model was trained and produced an individual risk score. Based on the median score of 0.2657412, patients in the training set were segregated into high- and low-risk subgroups (n = 175 each). A distribution plot (red/blue dots representing deceased/surviving patients, respectively) visualized a markedly greater mortality count within the high-risk subgroup (**Figure 1D**). Consistently, a heatmap demonstrated upregulated expression of all three signature genes in high-risk individuals (**Figure 1E**), who also experienced significantly inferior OS outcomes (**Figure 1F**). The model’s predictive strength was affirmed by the area under the curve (AUC) values for 1-, 3-, and 5-year survival, which measured 0.837, 0.841, and 0.840, exceeding the 0.6 benchmark across all time points (**Figure 1G**).

#### 3.1.2 Validation of the prognostic model

Model performance was further validated using an independent verification set. Applying the established algorithm, a risk score was computed for each patient, who were then dichotomized by the new median score (0.2973844) into high- and low-risk groups (n = 75 per group). Mirroring results from the training set, the high-risk group exhibited a greater death toll (**Figure 1H**), elevated expression of the three pivotal genes (**Figure 1I**), and significantly decreased OS (**Figure 1J**). The corresponding 1-, 3-, and 5-year survival AUC values in this validation cohort were 0.689, 0.600, and 0.632, all above 0.6, confirming the model’s robust generalizability (**Figure 1K**).

A comprehensive evaluation on the training set—utilizing confusion matrices, precision-recall (PR) curves, decision curve analysis (DCA), and calibration curves—demonstrated the model’s high discriminative power, net clinical benefit across a wide probability threshold, and strong predictive accuracy (**Figures 1L–O**).

To ascertain the independence of the risk score from conventional clinical parameters, univariate Cox regression on the training set identified the risk score, overall stage, T stage, and N stage as significant prognosticators (P < 0.05; **Figure 1P**). These four variables were subsequently entered into a multivariate Cox model after verifying the proportional hazards assumption. The multivariable analysis established the risk score as an independent predictor of patient survival (**Figure 1Q**).

In summary, the constructed RPL-centric prognostic model displays considerable accuracy and specificity in forecasting LUAD patient outcomes, though prospective validation in clinical cohorts remains a requisite next step.

### 3.2 Tumor mutation burden across defined risk subgroups

Mutational patterns were contrasted between high- and low-risk patients using the training set data. Genomic alterations were processed and visualized using the R package maftools, generating summary statistics and a waterfall plot (oncoplot) for each subgroup.

**Figure 2:**
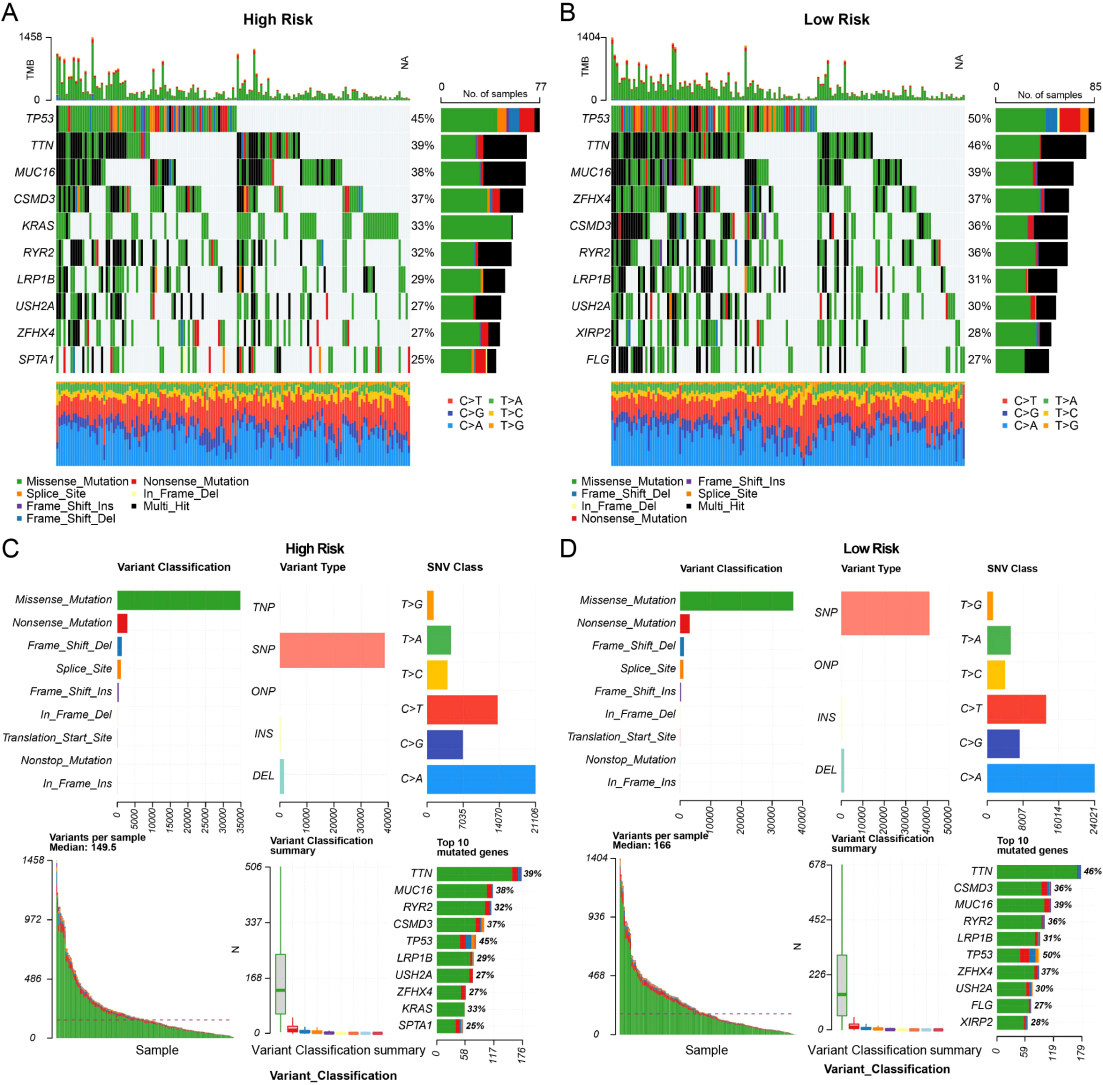
Assessment of tumor mutational burden across risk-defined subgroups. **(A, B)** Oncoplot (waterfall diagram) summarizing the landscape of somatic mutations in patients stratified by risk. **(C, D)** Summary of variant classification and mutation counts for high- and low-risk groups.

As depicted in **Figures 2A, B**, C>A transversions constituted the predominant single nucleotide variant class in both subgroups, aligning with established LUAD mutation signatures (23). The high-risk group’s ten most frequently mutated genes were TP53 (45%), TTN (39%), MUC16 (38%), CSMD3 (37%), KRAS (33%), RYR2 (32%), LRP1B (27%), USH2A (27%), ZFHX4 (27%), and SPTA1 (25%). In contrast, the top mutated genes in the low-risk group included TP53 (50%), TTN (46%), MUC16 (37%), ZFHX4 (31%), CSMD3 (31%), LRP1B (31%), USH2A (28%), XIRP2 (27%), and FLG (27%). Variant-per-sample statistics showed a modestly higher median mutation count in the low-risk cohort (median: 166 vs. 149.5 in the high-risk cohort); however, this difference was not statistically significant (Wilcoxon rank-sum test, P = 0.591; **Figures 2C, D**). A notable finding was the enrichment of KRAS mutations, observed in 33% of high-risk patients (**Figure 2C**), whereas this driver gene did not rank among the top ten in the low-risk group (**Figure 2D**). These results suggest that while both subgroups share recurrent oncogenic mutations (TP53, TTN, MUC16, CSMD3), the heightened prevalence of KRAS mutations in the high-risk subgroup may be a contributory factor to its adverse prognosis.

### 3.3 Comparative analysis of immune infiltration and escape across risk groups

**Figure 3:**
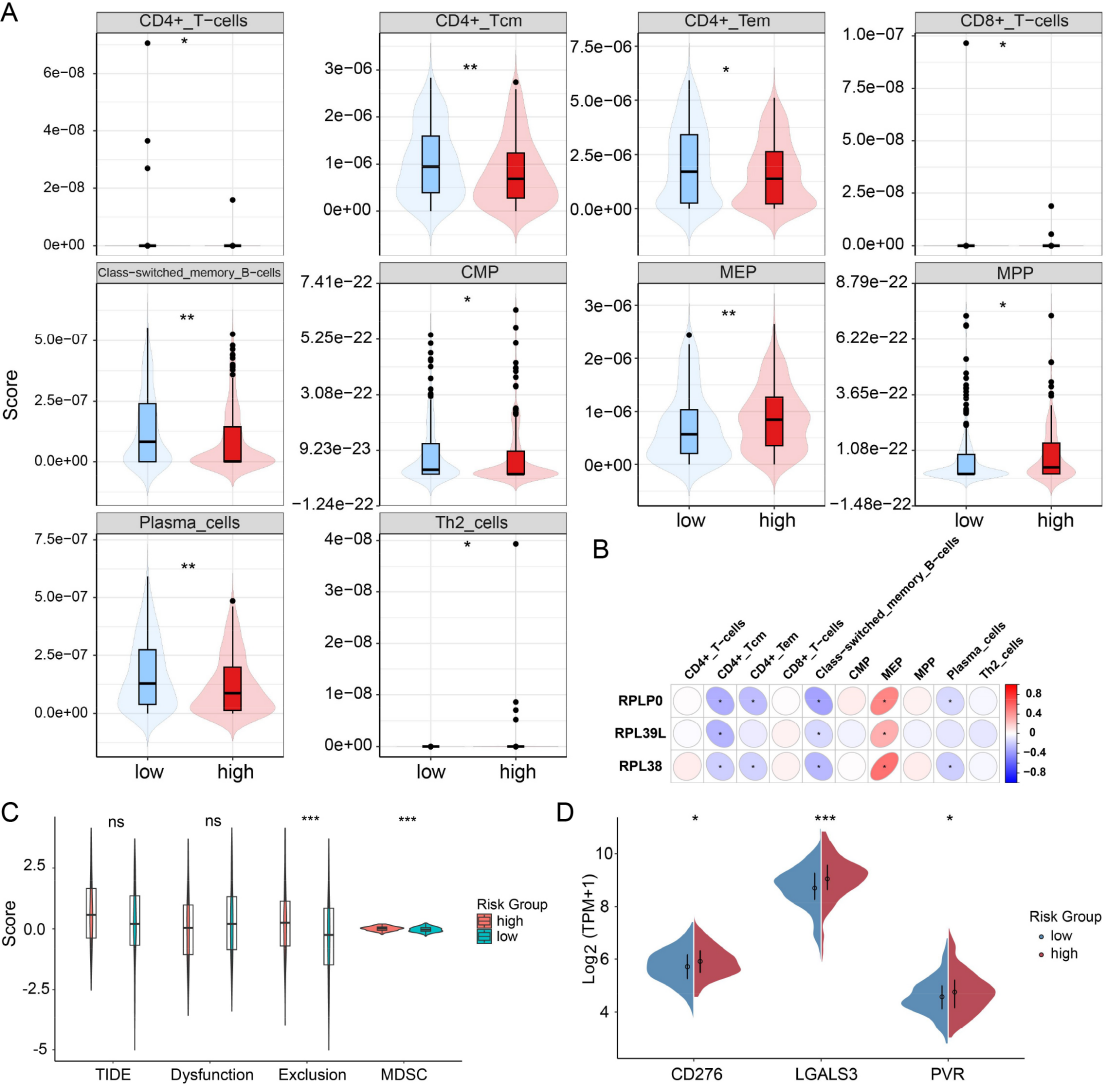
Immune infiltration landscape and escape features in high- versus low-risk LUAD patients. **(A)** Comparative abundance of tumor-infiltrating immune cell subsets between risk-stratified groups. **(B)** Correlation heatmap evaluating associations between the three prognostic RPL genes and differentially abundant immune cells. **(C)** Violin plots comparing TIDE, immune dysfunction, exclusion, and MDSC scores across risk categories. **(D)** Differential expression analysis of selected immune checkpoint molecules between high- and low-risk patients. ns *P*>0.05, *P < 0.05, **P < 0.01, *** *P* < 0.001. Statistical comparisons were performed using the Wilcoxon rank-sum test, as the data did not meet assumptions for parametric testing (assessed by Shapiro-Wilk and Levene’s tests).

The tumor microenvironment (TME) in LUAD is characterized by substantial immune heterogeneity, which critically influences disease progression (24). To assess differences in immune contexture, we applied the “xCell” algorithm to estimate immune cell infiltration levels within the training cohort. Ten immune cell subsets displayed significant variation between risk groups, including CD4^+^ T-cells, CD4^+^ Tcm, CD4^+^ Tem, CD8^+^ T-cells, class-switched memory B-cells, CMP, MEP, MPP, plasma cells, and Th2 cells (**Figure 3A**). Subsequent correlation analysis indicated that RPLP0 expression inversely correlated with infiltration of CD4^+^ Tcm and class-switched memory B-cells, and was positively linked with MEP cells. RPL39L levels showed negative associations with CD4^+^ Tcm, CD4^+^ Tem, class-switched memory B-cells, and plasma cells, while correlating positively with MEP. Similarly, RPL38 expression was negatively associated with CD4^+^ Tcm and positively with MEP cells (**Figure 3B**).

Given the critical role of immune escape in tumor evasion (25, 26), we computed Tumor Immune Dysfunction and Exclusion (TIDE) metrics. The high-risk group exhibited significantly elevated immune exclusion and myeloid-derived suppressor cell (MDSC) scores. Although TIDE scores were marginally higher in this group, the difference did not reach statistical significance (**Figure 3C**). Moreover, expression of immune checkpoint genes—CD276, LGALS3, and PVR—was markedly upregulated in high-risk patients (**Figure 3D**; **Supplementary Figure S2**). These findings imply an immunosuppressive TME in high-risk LUAD, though further experimental confirmation is warranted.

### 3.4 Drug sensitivity profiling between risk subgroups

Given the pronounced differences in immune infiltration patterns and immune escape features observed between risk groups, we further investigated whether these immunological disparities might translate into differential therapeutic vulnerabilities. We next evaluated differential pharmacosensitivity between risk groups by predicting response scores for 198 chemotherapeutic and targeted agents. Using Wilcoxon rank-sum tests (P < 0.05), 92 compounds showed significantly divergent efficacy between subgroups (**Supplementary Figures S3–S6**). Individuals in the low-risk category exhibited consistently lower IC50 scores across these agents, indicating heightened susceptibility to these treatments.

### 3.5 Multi-omics and spatial profiling of RPL38 expression

Among the three RPL genes comprising the prognostic signature, RPL38 was prioritized for in-depth study due to its elevated hazard ratio, strong statistical significance, and previously undefined role in LUAD. Pan-cancer screening revealed pronounced RPL38 upregulation across numerous malignancies (**Supplementary Figures S7A–C**). Elevated RPL38 expression correlated with adverse overall survival not only in LUAD but also in ACC, HNSC, KIRP, LIHC, and UVM (**Supplementary Figure S7D**). Mutation analysis indicated that RPL38 was most frequently altered in breast cancer (**Supplementary Figure S8A**), and CNV analysis identified recurrent RPL38 amplifications in KIRP, BLCA, and LIHC (**Supplementary Figure S8B**). A strong positive association between RPL38 copy number and mRNA expression was observed in BRCA and LUSC (**Supplementary Figure S8C**). DNA hypermethylation of RPL38 was inversely correlated with its transcript levels in PRAD, CHOL, LUSC, and others (**Supplementary Figure S9A**). Promoter methylation also negatively regulated RPL38 expression in PRAD, HNSC, LGG, and LUSC (**Supplementary Figure S9B**). These integrative omics analyses nominate RPL38 as a putative pan-cancer prognostic biomarker.

**Figure 4:**
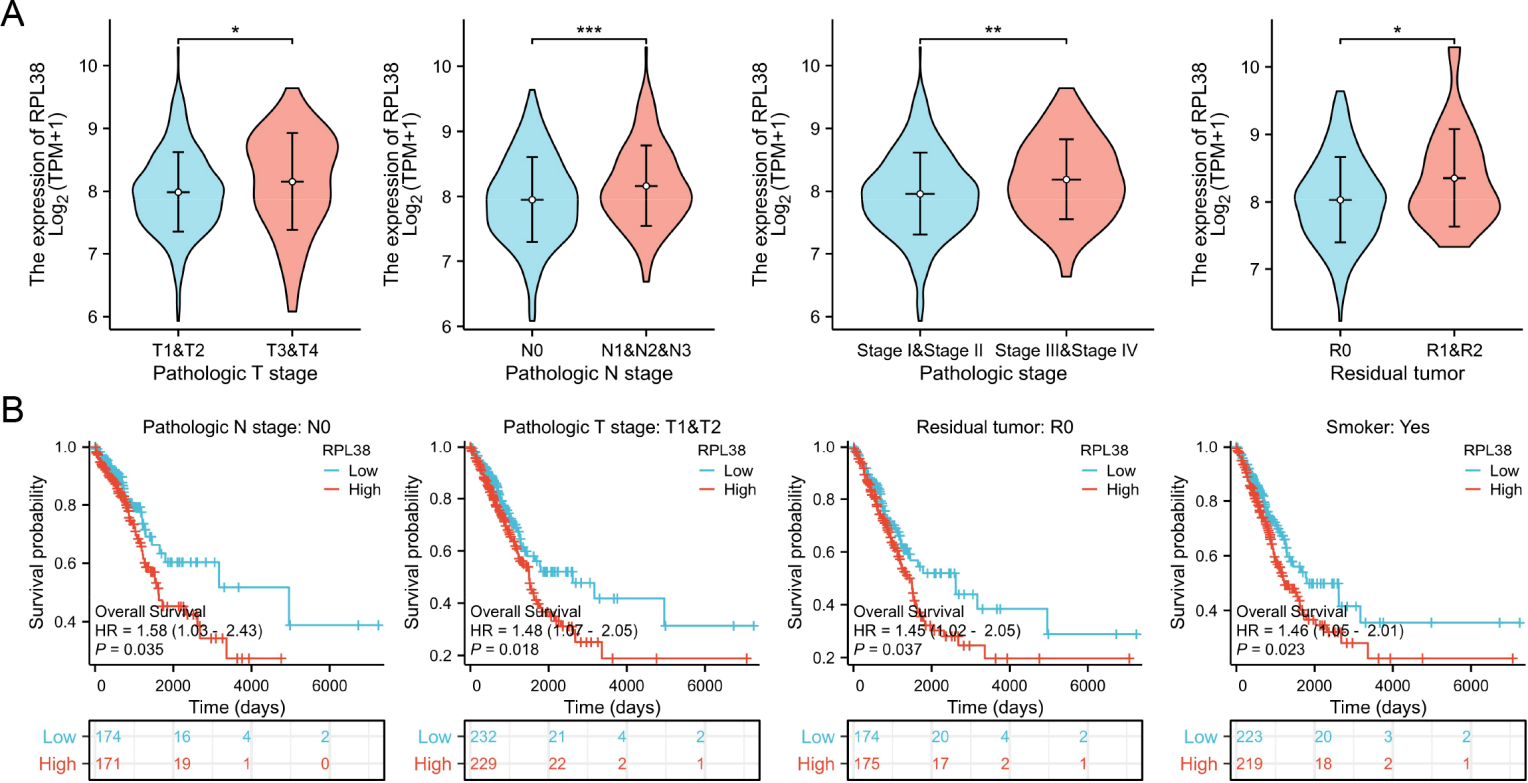
Relationship between RPL38 expression and clinical characteristics or survival in LUAD. **(A)** Association of RPL38 mRNA levels with various clinicopathological variables. **(B)** Survival curves based on Kaplan–Meier analysis stratified by high or low RPL38 expression. *P < 0.05, **P < 0.01, ***P < 0.001. Group comparisons for continuous variables in **(A)** were conducted using the Wilcoxon rank-sum test due to non-normal data distribution.

Within LUAD, RPL38 expression was significantly associated with clinicopathological variables including T stage, N stage, overall stage, and residual tumor (**Figure 4A**). Stratified survival analysis further linked high RPL38 expression to poor outcomes in subgroups such as N0, T1/T2, R0-resected, and smoking patients (**Figure 4B**).

**Figure 5:**
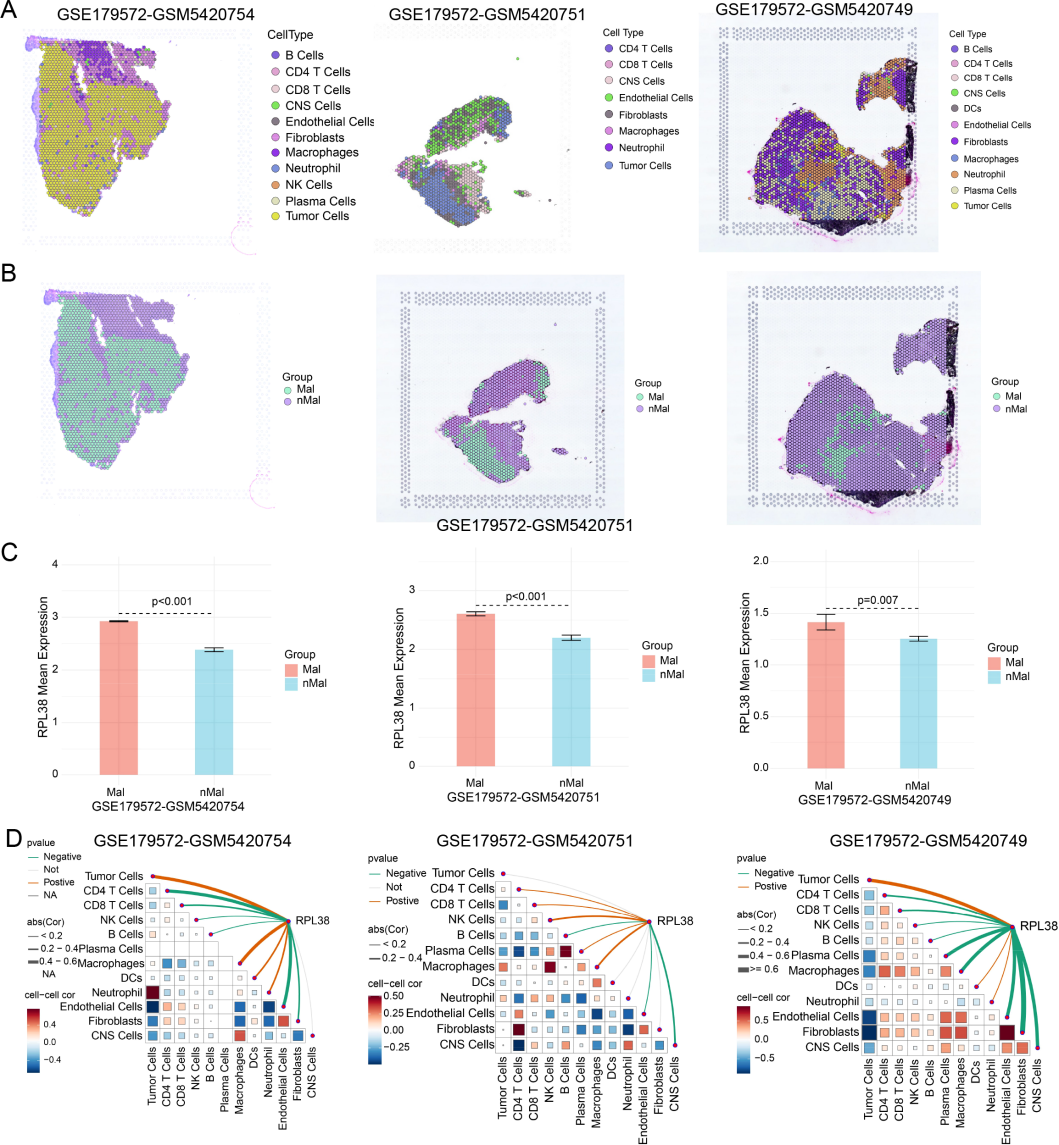
Spatial mapping of RPL38 in LUAD tissues. **(A)** Spatially resolved transcriptomic maps from three representative LUAD cases (GSE179572). **(B)** Annotated tissue regions highlighting tumor versus adjacent non-neoplastic areas. **(C)** Bar plot showing the average level of RPL38 expression within malignant regions. **(D)** Spearman correlation between RPL38 transcript abundance and tumor cell proportion across spatially defined spots.

To dissect spatial expression patterns, we analyzed transcriptomic data from LUAD tissue sections. RPL38 was significantly overexpressed in tumor regions relative to non-malignant areas, with higher mean expression in neoplastic zones (**Figures 5A–C**). Spearman correlation confirmed a positive association between RPL38 levels and tumor cell proportion within spatially resolved spots (**Figure 5D**), underscoring its role in tumor-specific expression within the TME.

### 3.6 Association between RPL38 and immune infiltration in LUAD

**Figure 6:**
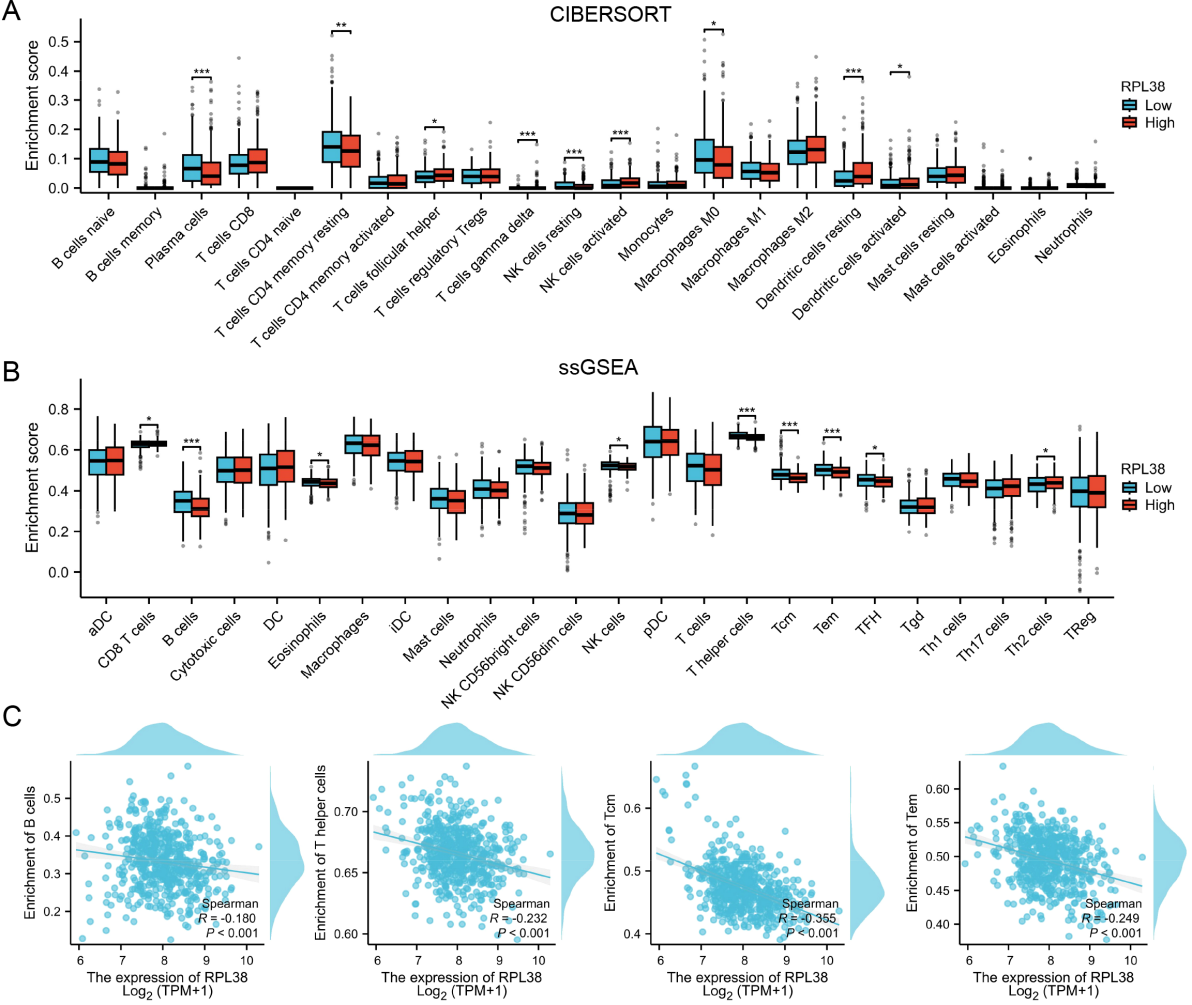
Immune infiltration landscape associated with RPL38 expression in LUAD. **(A, B)** Evaluation of immune cell abundance between RPL38-high and RPL38-low subgroups using CIBERSORT and ssGSEA algorithms. **(C)** Association analysis between RPL38 levels and enrichment scores for B cells, Th cells, and central/effector memory T cells (Tcm/Tem). ns *P*>0.05, *P < 0.05, **P < 0.01, ***P < 0.001. The Wilcoxon rank-sum test was applied for all comparisons, as the immune cell scores were not normally distributed.

To investigate how RPL38 relates to the immune landscape in LUAD, the TCGA-LUAD cohort was split into high- and low-expression subgroups. The low-RPL38 group displayed increased infiltration of plasma cells and resting CD4^+^ memory T cells, which may contribute to their better survival prognosis (**Figure 6A**). Single-sample gene set enrichment analysis (ssGSEA) further elucidated distinct immune functional states and cell-type composition between subgroups (**Figure 6B**). RPL38 levels were inversely associated with enrichment scores for B cells, T helper cells, and central/effector memory T cells (Tcm/Tem) (**Figure 6C**). Collectively, these observations suggest that RPL38 may modulate immune infiltration and shape the tumor microenvironment in LUAD.

### 3.7 Functional enrichment analysis of RPL38 in LUAD

**Figure 7:**
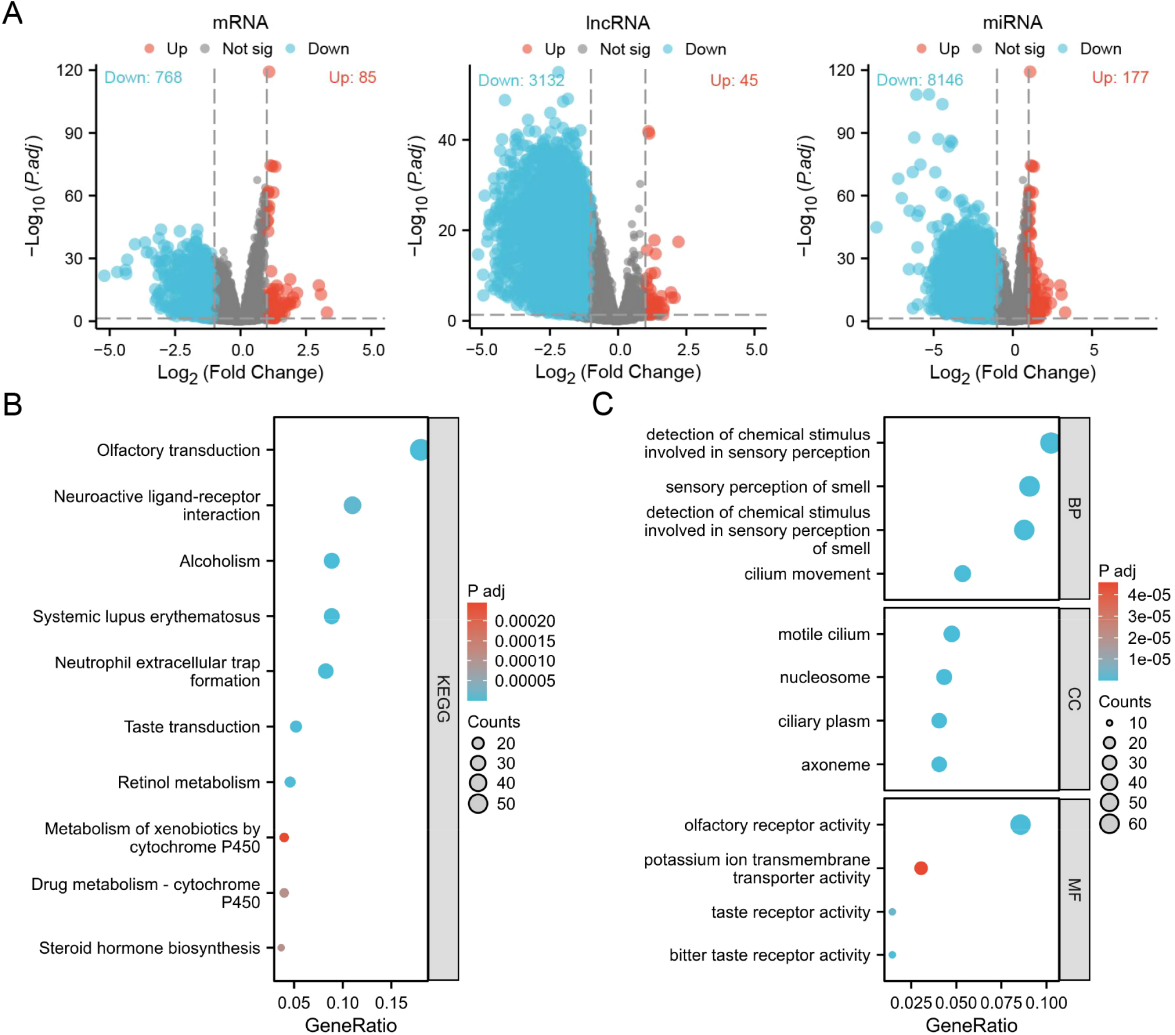
Differential expression and functional enrichment analysis based on RPL38 levels in LUAD. **(A)** Volcano plots visualizing differentially expressed mRNAs, lncRNAs, and miRNAs between RPL38-high and RPL38-low subgroups. **(B, C)** KEGG pathway and Gene Ontology enrichment analyses performed using the identified differentially expressed genes.

To uncover the biological functions of RPL38, TCGA-LUAD samples were divided into high- and low-expression groups (top and bottom 50%, respectively). Differential expression analysis (|log_2_FC| > 1, adjusted *P* < 0.05) identified 85 upregulated and 768 downregulated mRNAs, 45 upregulated and 3,132 downregulated lncRNAs, and 177 upregulated and 8,146 downregulated miRNAs (**Figure 7A**). KEGG pathway enrichment highlighted involvement in olfactory transduction, systemic lupus erythematosus, alcoholism, neutrophil extracellular trap formation, and taste transduction (**Figure 7B**). Gene Ontology (GO) analysis further linked these genes to biological processes such as detection of chemical stimuli in sensory perception, sensory perception of smell, and nucleosome assembly (**Figure 7C**).

**Figure 8:**
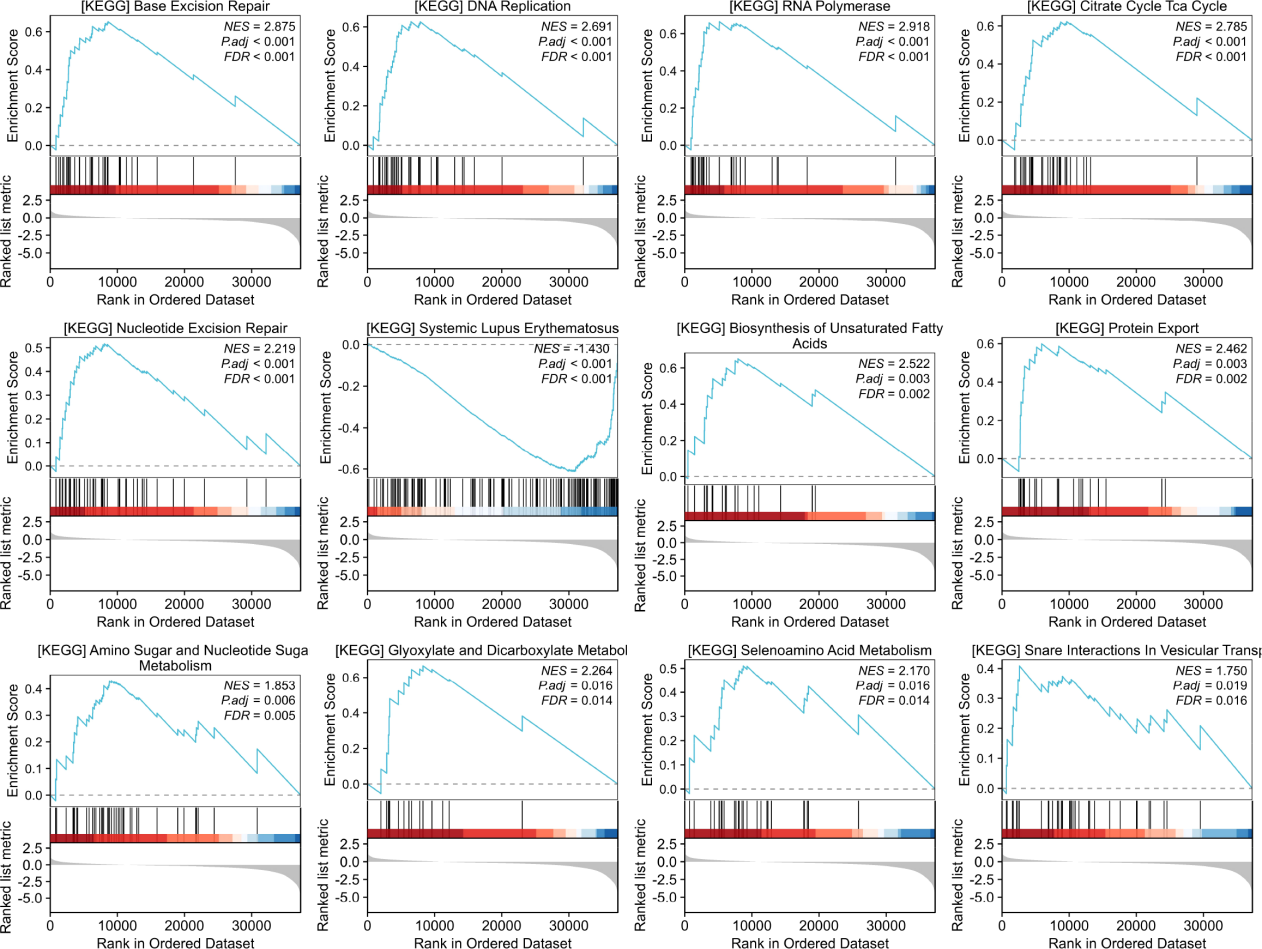
GSEA enrichment RPL38-related signaling pathway in LUAD.

GSEA revealed that RPL38 expression was positively correlated with pathways including base excision repair, DNA replication, RNA polymerase activity, the citrate (TCA) cycle, nucleotide excision repair, systemic lupus erythematosus, biosynthesis of unsaturated fatty acids, protein export, amino sugar and nucleotide sugar metabolism, glyoxylate and dicarboxylate metabolism, selenoamino acid metabolism, and SNARE interactions in vesicular transport (**Figure 8**).

### 3.8 RPL38 knockdown attenuates malignant behaviors of LUAD Cells

**Figure 9:**
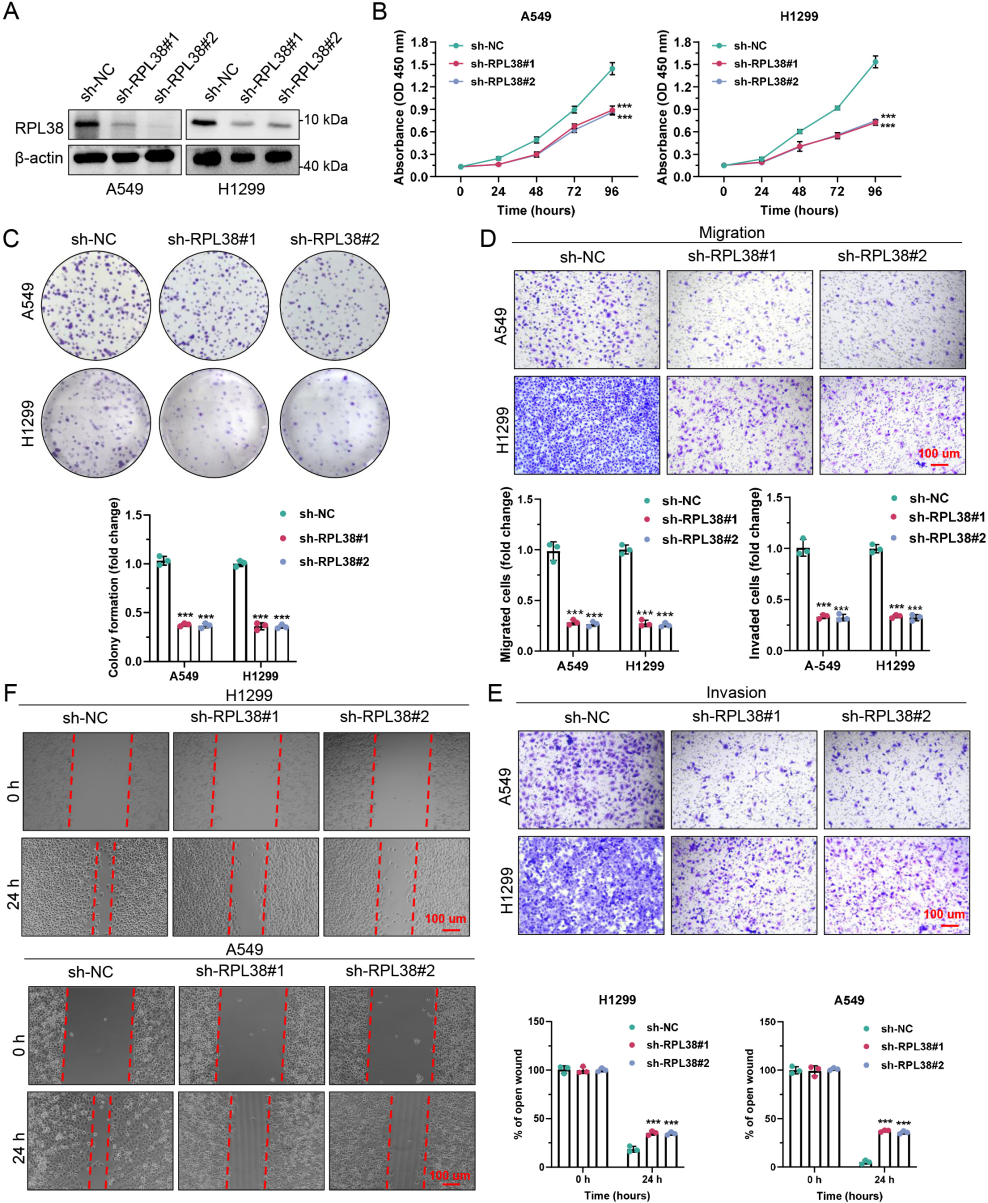
Oncogenic function of RPL38 in LUAD pathogenesis. **(A)** Immunoblot validation of RPL38 knockdown in A549 and H1299 cells transduced with shRNA targeting RPL38. **(B)** Cell viability measured by CCK-8 assay at specified intervals after RPL38 silencing. **(C)** Colony formation assay showing reduced clonogenicity in RPL38-depleted cells. **(D, E)** Transwell assays quantifying migration and invasion capacities after RPL38 ablation. **(F)** Delayed wound closure upon RPL38 knockdown, as assessed by scratch assay. Scale bars: 100 μm. Data represent mean ± SD from three biological replicates. ***P < 0.001. Statistical significance was determined by two-tailed Student’s t-test, as the data met assumptions of normality and equal variance.

Given the elevated expression of RPL38 in LUAD, we stably knocked down RPL38 in A549 and H1299 cell lines using lentiviral shRNA. Western blotting confirmed efficient RPL38 depletion in both lines (**Figure 9A**). CCK−8 assays demonstrated significantly reduced cell viability following RPL38 knockdown (**Figure 9B**). Colony formation assays also showed decreased clonogenic capacity in RPL38-depleted cells (**Figure 9C**).

To assess the role of RPL38 in cell motility, Transwell migration/invasion assays and scratch wound healing experiments were performed. RPL38 knockdown markedly reduced both migratory and invasive potential compared with control cells (**Figures 9D–E**). Similarly, wound closure was significantly delayed upon RPL38 depletion (**Figure 9F**). Together, these *in vitro* findings indicate that silencing RPL38 impairs the proliferative, migratory, and invasive capacities of LUAD cells.

### 3.9 RPL38 depletion inhibits tumor growth *In Vivo*

**Figure 10:**
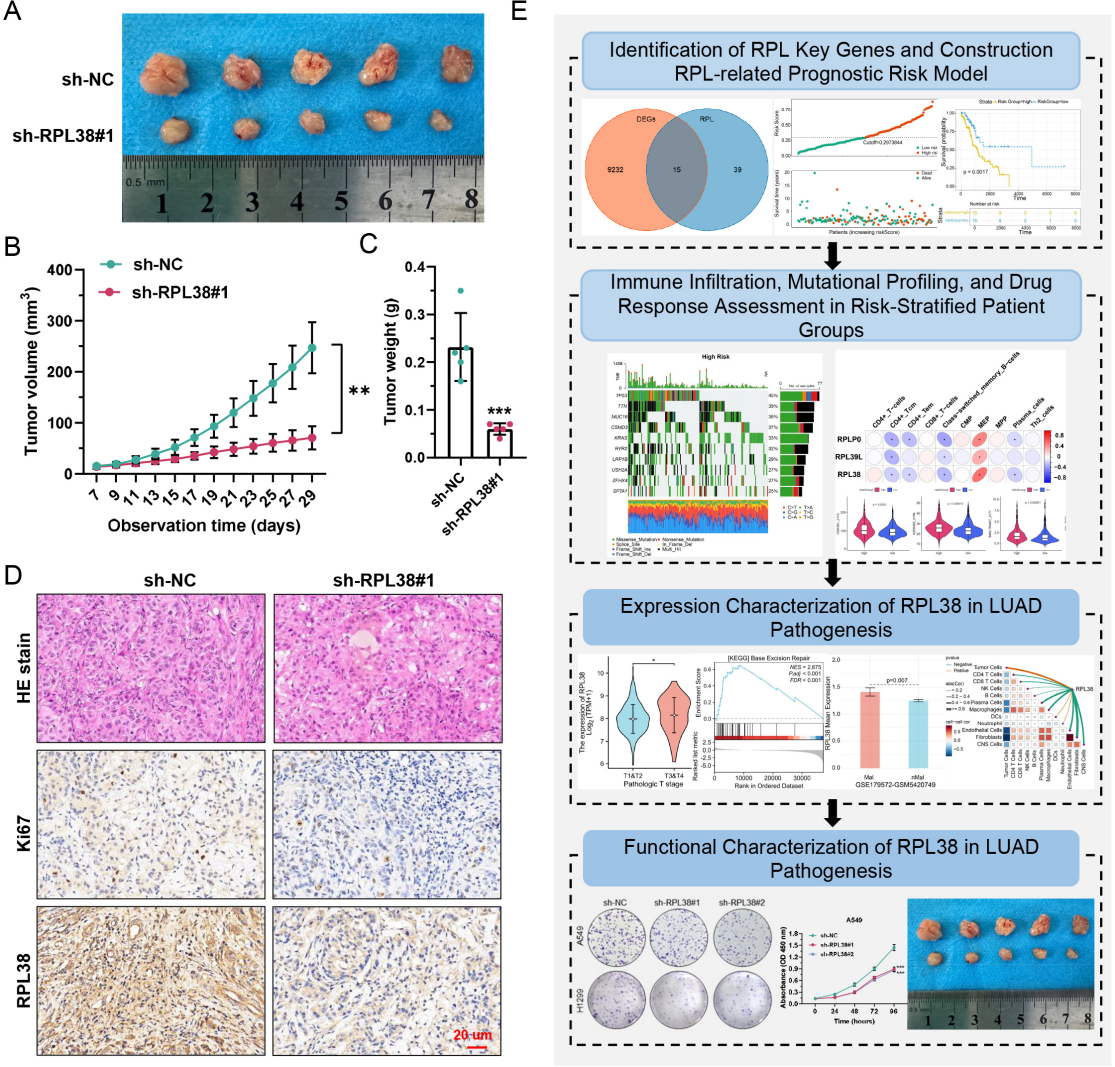
*In vivo* suppression of LUAD xenograft growth by RPL38 depletion. **(A)** Representative photograph of excised tumors at day 29 post-implantation. **(B)** Growth kinetics of subcutaneous tumors over time. **(C)** Final tumor weights measured on day 29 and presented as a bar graph. **(D)** Immunohistochemical staining for Ki−67 and RPL38 in tumor sections. Scale bars: 20 μm. **(E)** Schematic summary illustrating the principal findings of this study. ** *P* < 0.01, *** *P* < 0.001. Tumor volume and weight comparisons were analyzed using Student’s t-test.

The *in vivo* function of RPL38 was evaluated using subcutaneous xenograft models. Mice injected with A549-sh-RPL38#1 cells exhibited significantly slower tumor growth over 29 days compared to those receiving control (sh-NC) cells, as reflected by reduced tumor volume progression and lower final tumor weights (**Figures 10A–C**). Immunohistochemistry confirmed decreased expression of both RPL38 and the proliferation marker Ki67 in knockdown-derived tumors (**Figure 10D**). A graphical model summarizing the main conclusions of this study is provided (**Figure 10E**). In summary, we identified key RPL genes in LUAD and constructed a prognostic model based on these markers. The differential expression, clinical relevance, and immune-modulatory role of RPL38 were systematically validated through multi−omics approaches. Functional assays *in vitro* and *in vivo* established the tumor-promoting function of RPL38 in LUAD pathogenesis.

## 4 Discussion

The ribosomal protein family, traditionally known for its central function in translation, is increasingly implicated in oncogenesis and tumor development (27, 28). Beyond their canonical role, specific ribosomal proteins can form specialized “oncoribosomes” that alter translational fidelity and preference, potentially driving tumorigenic programs. Here, we constructed and independently verified a prognostic signature for LUAD utilizing three RPL genes (RPLP0, RPL39L, and RPL38), which successfully categorized patients into risk strata with markedly different overall survival. Our integrative approach highlights not only the predictive utility of these RPL markers but also suggests their involvement in modulating the immune landscape and therapeutic susceptibility. Existing literature notes the prognostic relevance of RPLP0 in LUAD (29), the upregulation of RPL39L in HCC (30), and the potential of RPL38 as a biomarker in pancreatic cancer (31).

The developed model exhibited consistent robustness. High time-dependent AUCs in both derivation and validation sets, supported by favorable decision-curve and calibration metrics, underscore its reliable predictive capacity for LUAD outcomes. Importantly, multivariable analysis established the model’s risk score as an independent prognostic indicator, maintaining significance after adjustment for standard clinicopathological factors including TNM stage. This implies that the RPL signature encapsulates distinct biological determinants of tumor behavior beyond conventional staging. We note that this model is intended for molecular risk assessment alongside, not as a substitute for, established TNM staging (32).

Although the model demonstrated overall stable performance in the internal validation cohort, a modest decrease in the 3-year AUC (0.600) compared with the training set was observed. This borderline reduction is not unexpected and may reflect intrinsic cohort heterogeneity within the TCGA-LUAD dataset, including variations in clinical characteristics, treatment regimens, and follow-up duration between subsets. In addition, the relatively small number of long-term survival events may reduce discrimination accuracy at intermediate time points. Importantly, despite this slight attenuation, the model consistently stratified patients into distinct risk groups with significant survival differences, supporting its overall robustness and clinical relevance.

Mutational patterns were contrasted between high- and low-risk patients using the training set data. Interestingly, KRAS mutations were significantly enriched in the high-risk group, raising the possibility of a functional interplay between RPL38 expression and KRAS pathway activation. One plausible hypothesis is that elevated RPL38 may enhance KRAS-driven oncogenic signaling, either by modulating the translation of KRAS or its downstream effectors, or by influencing ribosome-mediated translational control of key pathway components. This potential synergism could contribute to the aggressive phenotype observed in high-risk patients. Future mechanistic studies, such as RPL38 knockdown or overexpression in KRAS-mutant cell lines followed by assessment of MAPK/ERK or PI3K/AKT pathway activity, could clarify this regulatory relationship and uncover potential therapeutic vulnerabilities.

An important finding from our analysis is the link between high-risk status and traits of an immunosuppressive microenvironment, corroborated by human spatial transcriptomic profiles (33, 34). We observed altered infiltration of specific T-cell and B-cell subsets in high-risk patients, concurrent with elevated expression of immune checkpoint genes (CD276, LGALS3, PVR) and higher TIDE and MDSC scores—features suggestive of active immune evasion (35, 36). It must be noted that the employed xenograft models, being immunodeficient, preclude direct *in vivo* assessment of immune compartment changes. Typically, cytotoxic T cells and memory B cells are considered antitumor effectors, whereas regulatory T cells and MDSCs promote escape. Although our multi-omics data implicate these RPL genes in immune regulation, definitive proof of causality—such as whether RPL38 knockdown alters specific immune populations—awaits investigation in immunocompetent or syngeneic models. Thus, we propose these RPL members as potential modulators of immunosuppressive pathways, pending further mechanistic validation.

Pharmacogenomic profiling further identified 92 agents with divergent predicted efficacy between risk groups. It should be noted that these IC50 values were inferred from computational models and are therefore subject to inherent limitations. Consequently, these results provide a prioritized candidate list for tailored therapy exploration rather than definitive therapeutic recommendations. Further validation in preclinical systems is essential to confirm their actual efficacy. Notably, the low-risk subgroup demonstrated a trend toward greater sensitivity to several compounds, possibly reflecting a less immune-suppressive milieu or distinct tumor biology. These results resonate with recent work on ribosomal components in NSCLC immunity and immunotherapy response (37).

Focusing on RPL38—chosen for its strong hazard ratio and previously undefined role in LUAD—revealed its wider relevance as a putative pan-cancer driver. Its recurrent overexpression in diverse tumors, correlation with adverse outcomes in six cancer types, and connection to CNV and DNA methylation changes underscore its fundamental oncogenic role. While its upregulation is observed across cancers, our data establish its particular significance in LUAD through its strong prognostic power, specific association with KRAS mutations, and its link to a clinically relevant immunosuppressive microenvironment in this disease context. Spatial transcriptomics unequivocally confirmed its tumor-enriched expression at cellular resolution, solidifying its pathogenic importance in LUAD.

This study has several constraints. First, while internal validation used an independent TCGA cohort, external confirmation with prospective, multi-center datasets is needed to establish generalizability. Second, the model prioritizes genetic signature prediction; we did not adjust for clinical covariates during validation, which may affect clinical interpretability. Future iterations could integrate clinical features using multivariate Cox, competing-risk, or multimodal modeling. Third, the precise mechanisms by which RPL38 and related genes foster immune suppression and progression are not fully defined. However, our integrated analysis provides a clear roadmap for future investigation. The strong association between high RPL38 expression, KRAS mutation enrichment, and an immunosuppressive microenvironment generates two key, testable hypotheses (1): that RPL38 may preferentially modulate the translation efficiency of specific oncogenic transcripts (within the KRAS pathway), and (2) that it may directly or indirectly influence the expression of immune checkpoint molecules or the recruitment of immunosuppressive cells. Although bioinformatics linked them to immune infiltration and epigenetic regulation, direct experimental proof—how RPL38 affects T-cell function or checkpoint expression—is lacking. Finally, drug sensitivity profiles were computationally inferred; actual clinical responses may differ. Subsequent work should elucidate RPL-mediated immune mechanisms and test the model’s utility in prospective immunotherapy trials.

## 5 Conclusion

In summary, this research presents a robust prognostic model for LUAD founded on RPL gene expression, capturing dimensions of both intrinsic tumor malignancy and the surrounding immune contexture. This signature holds promise as a stratification instrument in clinical practice, potentially informing choices related to immunotherapy and chemotherapy. Additionally, we propose RPL38 as a new and viable pan-cancer biomarker and tumor-promoting factor. Subsequent investigations should aim to clarify the exact molecular pathways by which these RPL genes, especially RPL38, contribute to disease advancement and immune escape, and to assess their suitability for therapeutic intervention.

## Data Availability

The original contributions presented in the study are included in the article/**Supplementary Material**. Further inquiries can be directed to the corresponding author/s.
