# Supplementary material for: Decoding the role of RPL38 in lung adenocarcinoma: a multi-omics approach
Source: Front Immunol. 2026 Feb 12;17:1778481. doi: 10.3389/fimmu.2026.1778481 (PMC12935985; doi:10.3389/fimmu.2026.1778481)
Supplement: Supplementary file 14 [file Table1.docx]

**Decoding the Role of RPL38 in Lung Adenocarcinoma: A Multi-Omics Approach**


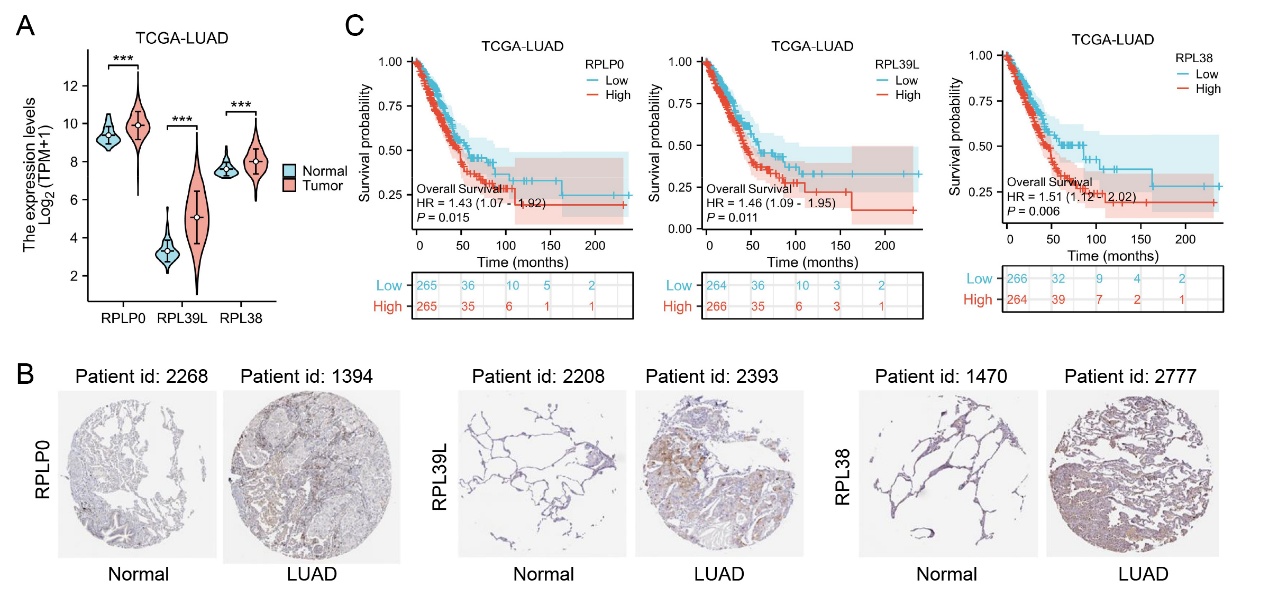


**Supplemental Figure 1. Comprehensive expression and prognostic analysis of three candidate genes in LUAD.**

(A) Transcriptional profiles of three candidate genes in the TCGA-LUAD cohort. (B) Immunohistochemical validation of protein expression patterns in LUAD using the Human Protein Atlas. (C) Kaplan–Meier survival analysis evaluating the prognostic significance of three candidate genes in LUAD. *** *P* < 0.001. Statistical significance in (C) was determined by the log-rank test.


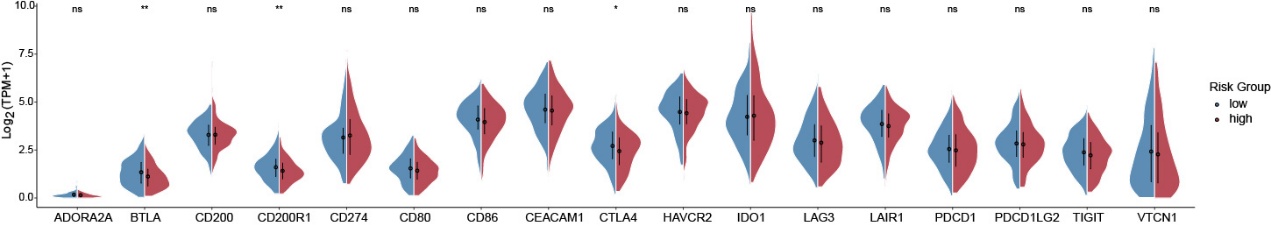


**Supplemental Figure 2. Expression levels of classic immune checkpoint molecules in high and low risk groups.** ns *P* > 0.05, **P* < 0.05, ** *P* <0.01. Differences were assessed using the Wilcoxon rank-sum test, as the expression data did not meet normality assumptions.

**
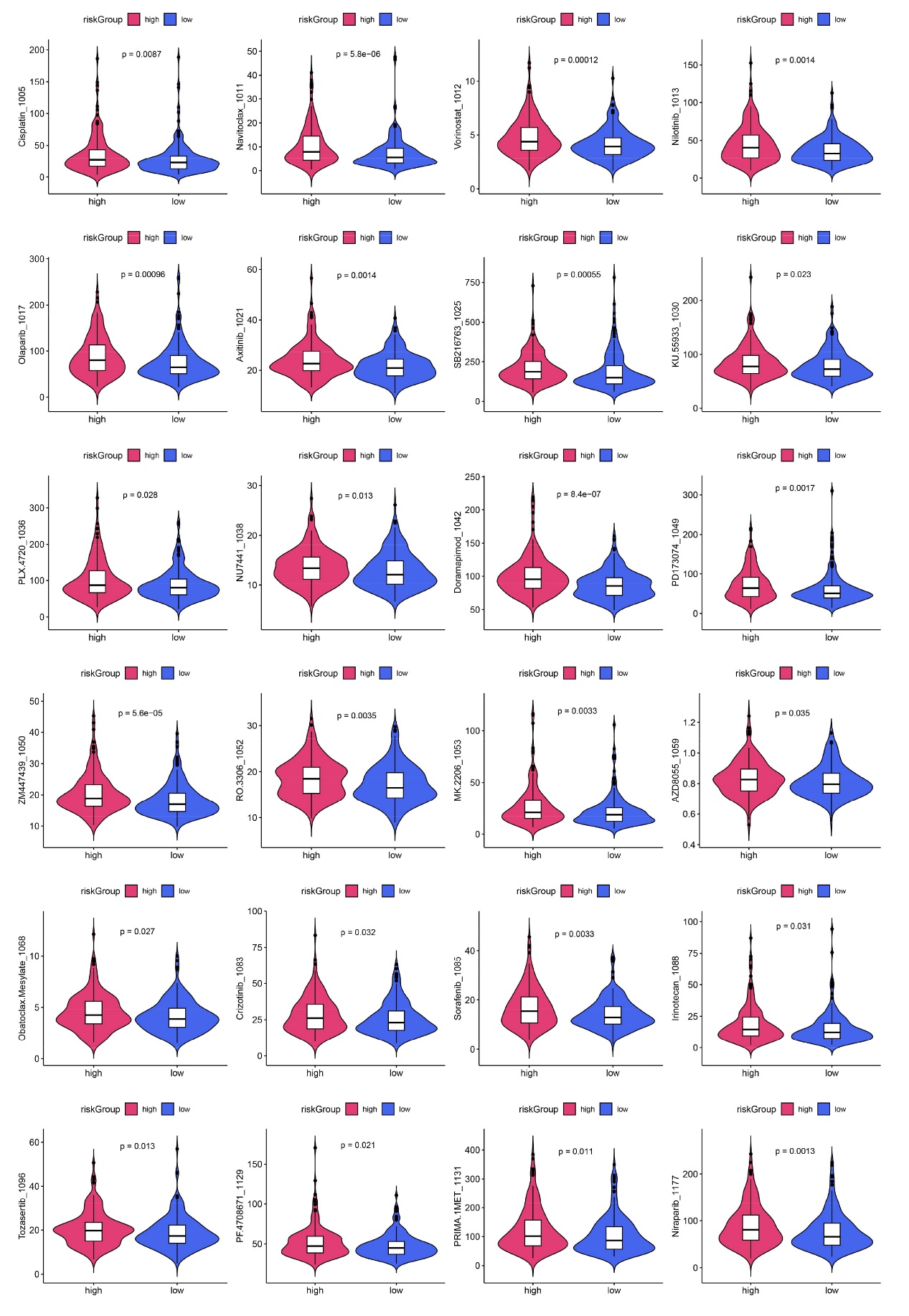
**

**Supplemental Figure 3. Sample chart of sensitivity scores of patients in high/low risk groups to common anti-tumor drugs.**


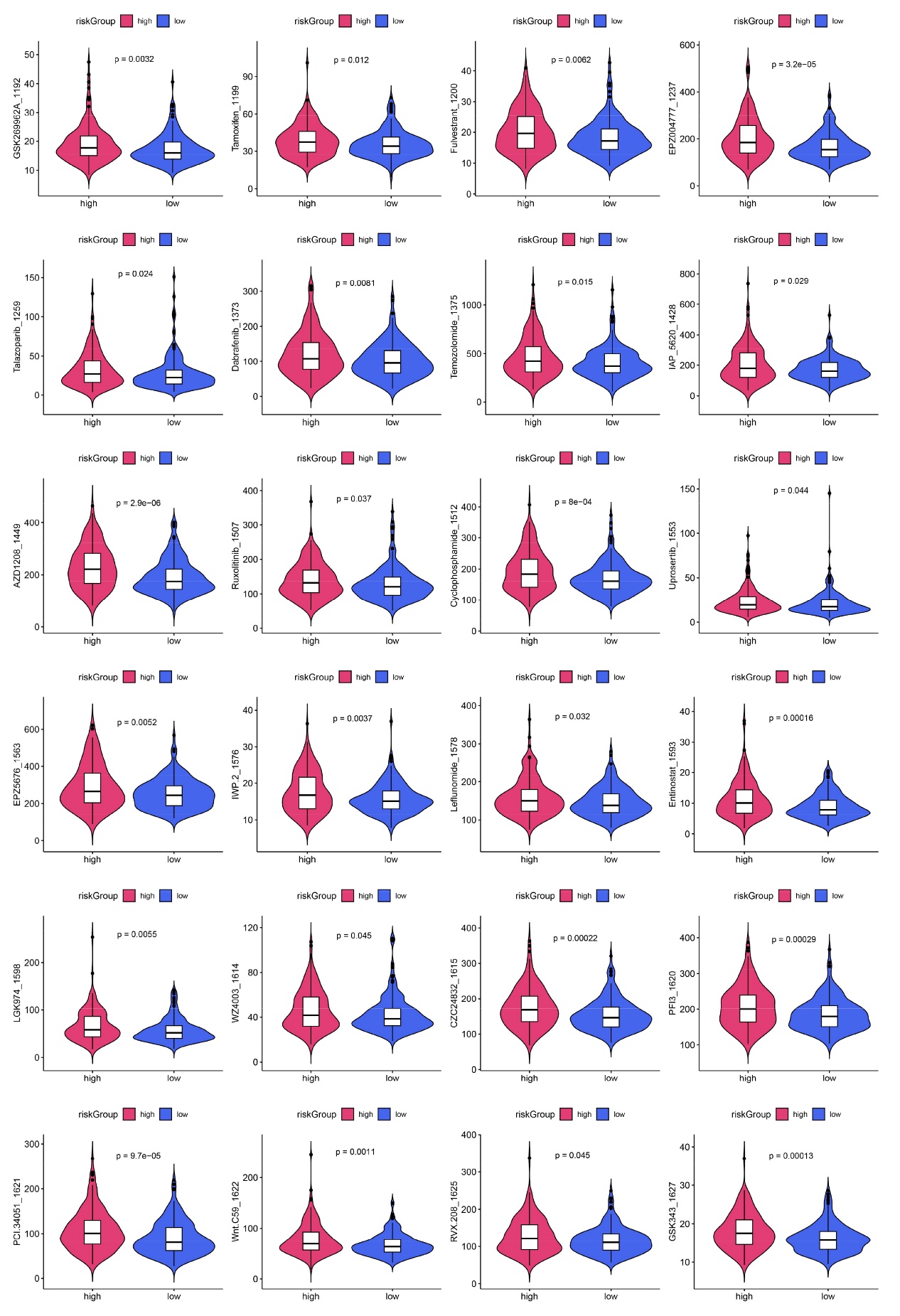


**Supplemental Figure 4. Sample chart of sensitivity scores of patients in high/low risk groups to common anti-tumor drugs.**


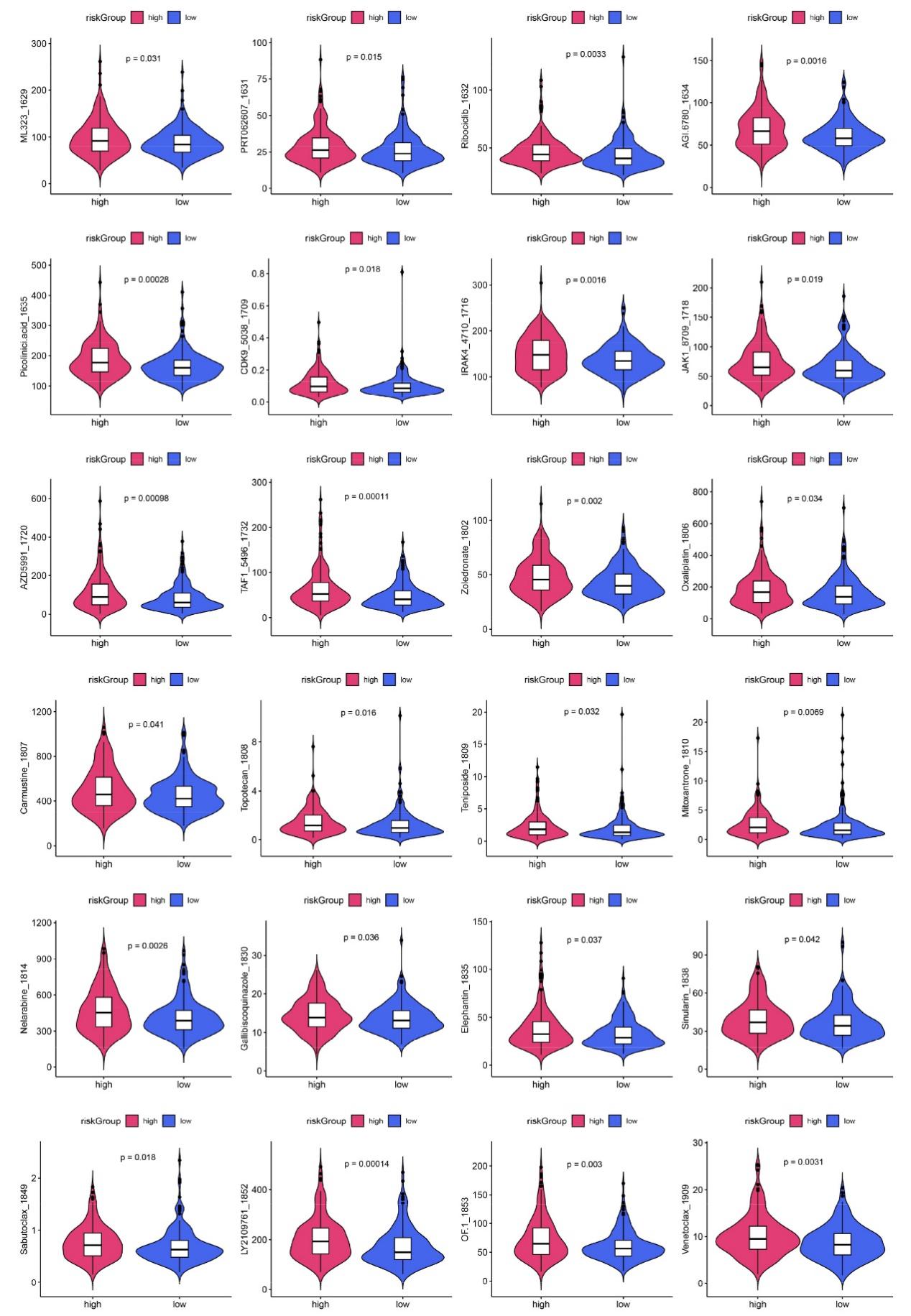


**Supplemental Figure 5. Sample chart of sensitivity scores of patients in high/low risk groups to common anti-tumor drugs.**


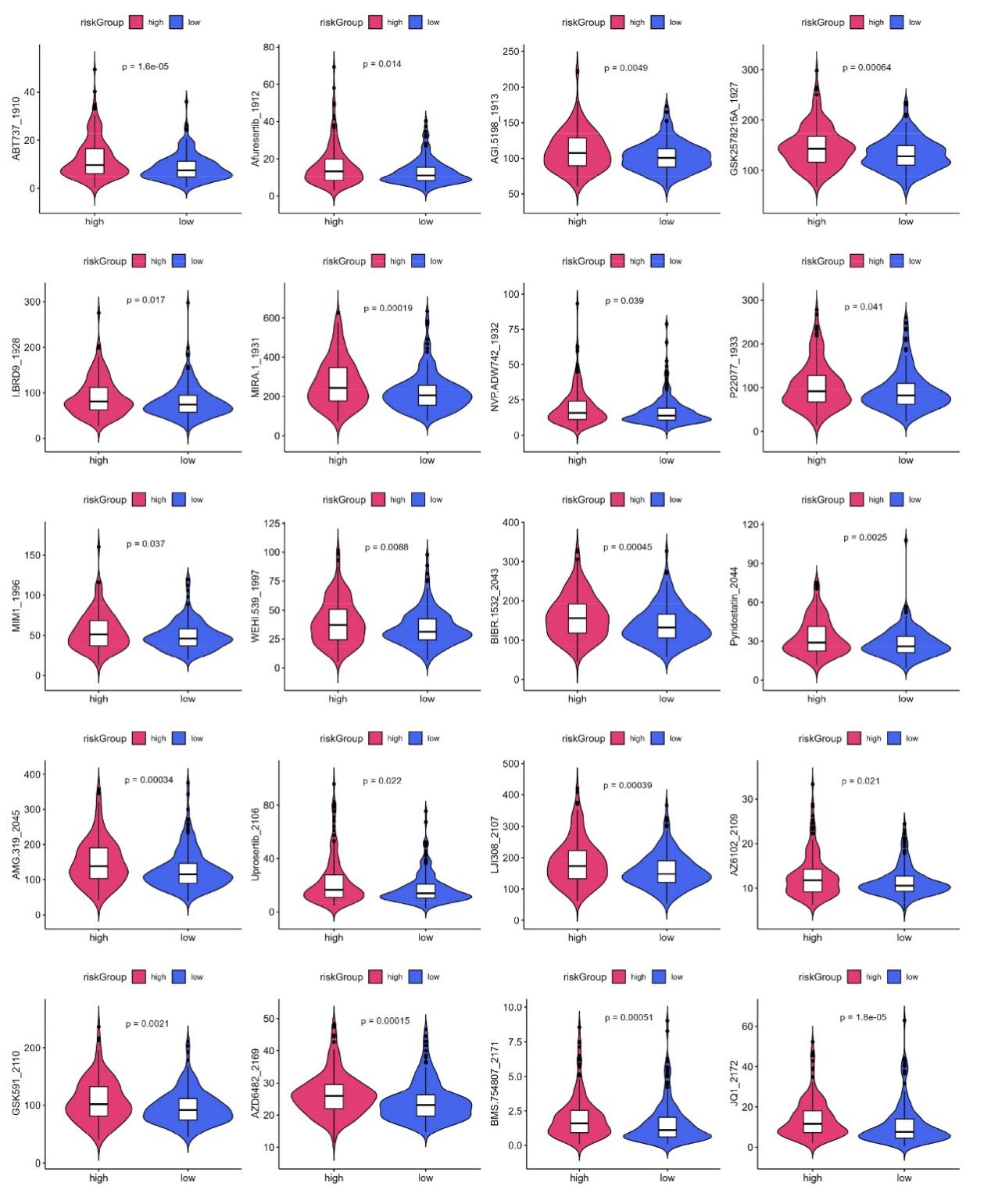


**Supplemental Figure 6. Sample chart of sensitivity scores of patients in high/low risk groups to common anti-tumor drugs.**


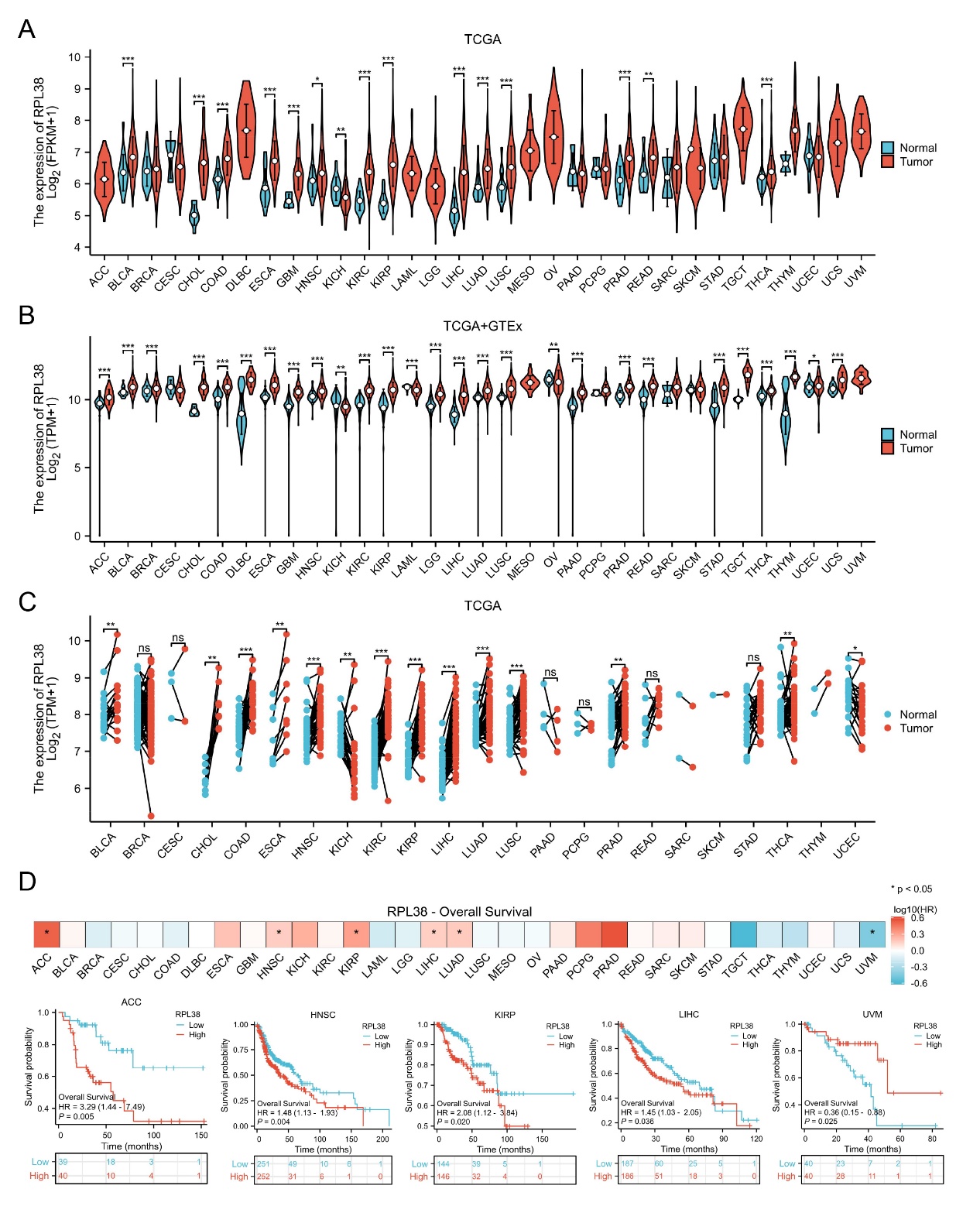


**Supplemental Figure 7. Pan-cancer analysis of RPL38 expression and its prognostic significance.**

(A) Differential RPL38 expression patterns across multiple cancer types based on TCGA data. (B) Comparative analysis of RPL38 expression in human malignancies using integrated TCGA and GTEx datasets. (C) Evaluation of RPL38 expression in tumor tissues versus matched adjacent normal tissues from TCGA. (D) Kaplan–Meier survival curves depicting the association between RPL38 expression and overall survival in patients with ACC, HNSC, KIRP, LIHC, or UVM. ns *P*>0.05, **P*<0.05, ** *P* <0.01, *** *P* <0.001. Group comparisons in (A-C) were performed using the Wilcoxon rank-sum test. Survival analysis in (D) was conducted using the log-rank test.


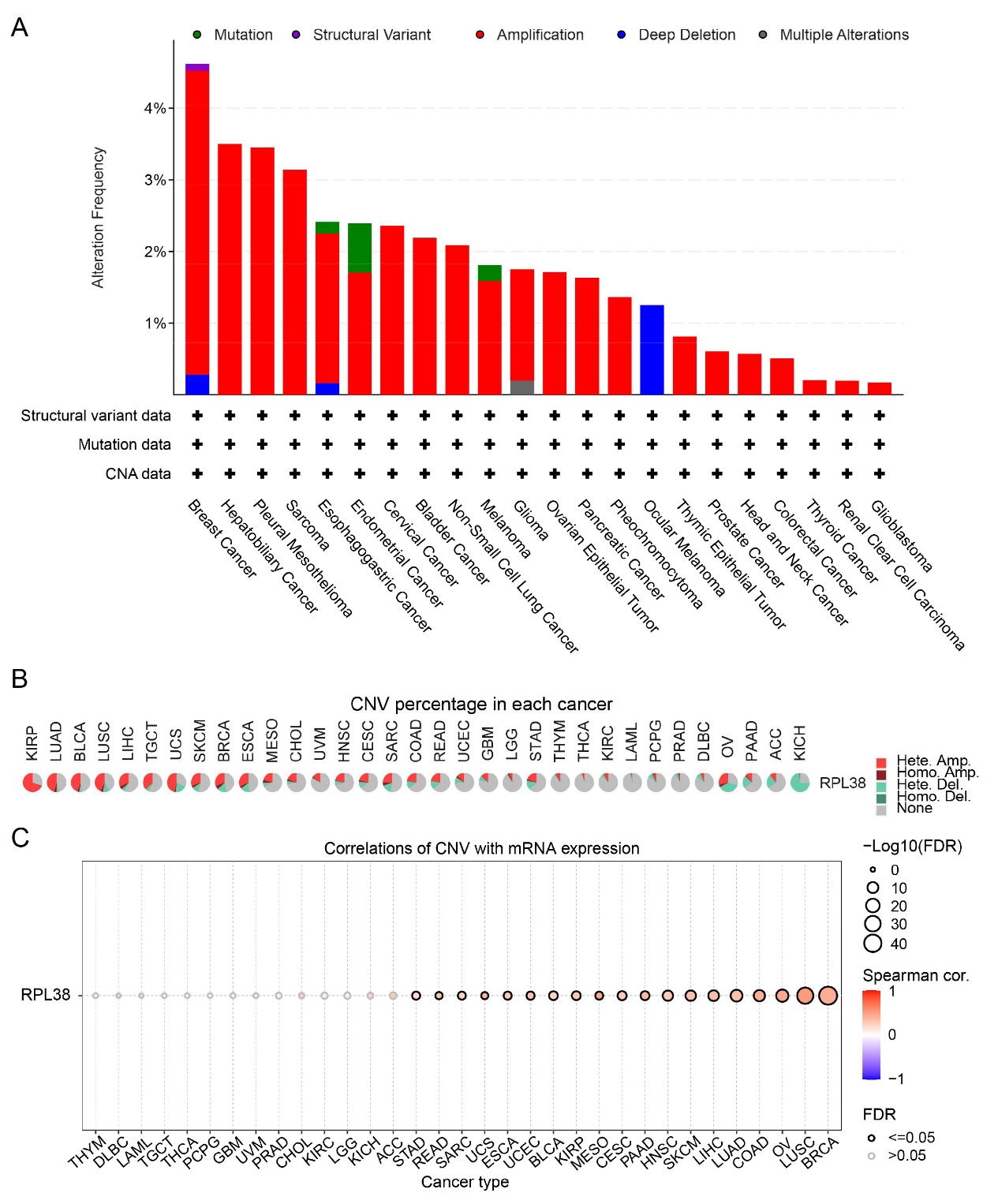


**Supplemental Figure 8. Mutational analysis of RPL38 in pancancer.**

(A) Distribution patterns and diversity of RPL38 mutation types in various malignancies. (B) Pan-cancer analysis of copy number variation (CNV) frequency for RPL38. (C) Relationship between RPL38 copy number alterations and corresponding mRNA expression levels. The correlation in (C) was evaluated using Spearman‘s method.

**
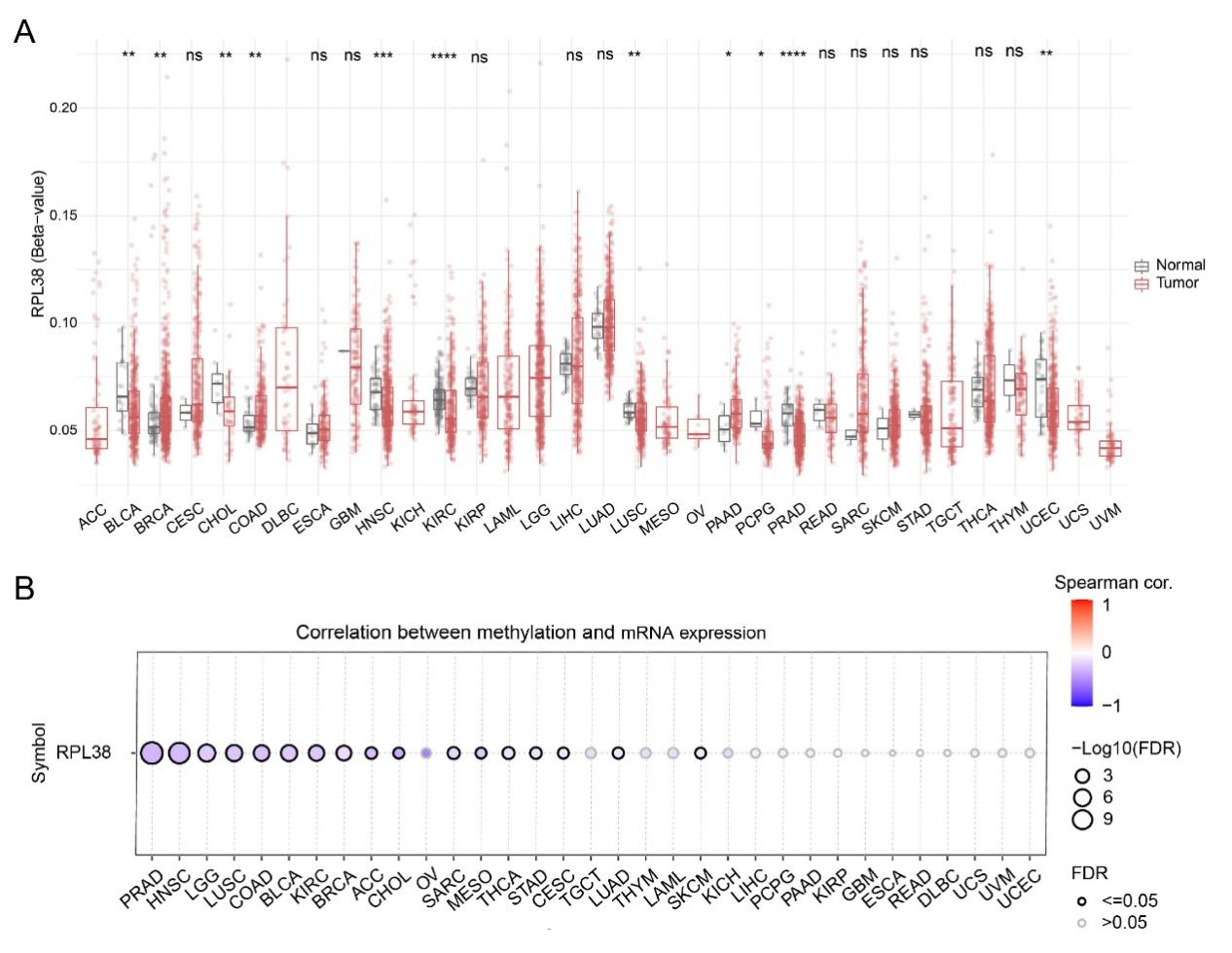
**

**Supplemental Figure 9.** **Comprehensive DNA methylation analysis of RPL38 in pan-cancer.**

(A) DNA methylation profiles of RPL38 across various cancer types. (B) Association between RPL38 expression levels and its promoter methylation status. ns *P* > 0.05, * *P* < 0.05, ** *P* < 0.01, *** *P* < 0.001. The association in (B) was analyzed using Spearman‘s correlation.
